# The Effect of Dietary Intervention With High-Oleocanthal and Oleacein Olive Oil in Patients With Early-Stage Chronic Lymphocytic Leukemia: A Pilot Randomized Trial

**DOI:** 10.3389/fonc.2021.810249

**Published:** 2022-01-21

**Authors:** Andrea Paola Rojas Gil, Ioannis Kodonis, Anastasios Ioannidis, Tzortzis Nomikos, Ioannis Dimopoulos, Georgios Kosmidis, Maria Efthymia Katsa, Eleni Melliou, Prokopios Magiatis

**Affiliations:** ^1^ Laboratory of Biology and Biochemistry, Department of Nursing, Faculty of Health Sciences, University of Peloponnese, Tripoli, Greece; ^2^ Hematology Department, General Hospital of Lakonia, Sparta, Greece; ^3^ Department of Nutrition and Dietetics, School of Health Sciences and Education, Harokopio University, Athens, Greece; ^4^ School of Management, University of Peloponnese, Kalamata, Greece; ^5^ Laboratory of Pharmacognosy and Natural Products Chemistry, Department of Pharmacy, National and Kapodistrian University of Athens, Athens, Greece

**Keywords:** extra virgin olive oil (EVOO), apoptosis, randomized trial, dietary intervention, oleocanthal, oleacein, Chronic Lymphocytic Leukemia (CLL)

## Abstract

**Aim:**

Oleocanthal and oleacein (OC/OL) have important *in vitro* and *in vivo* antitumor properties; however, there is no data about their anticancer activity in humans. The aim of this pilot study was to test if patients at early stage of chronic lymphocytic leukemia (CLL) could adhere to and tolerate an intervention with high OC/OL extra virgin olive oil (EVOO) and if this intervention could lead to any changes in markers related to the disease.

**Methods:**

A pilot dietary intervention (DI) was made in patients with CLL in Rai stages 0–II who did not follow any treatment (NCT04215367). In the first intervention (DI1), 20 CLL patients were included in a blind randomized study and were separated into two groups. One group (A) of 10 patients consumed 40 ml/day of high OC/OL-EVOO (416 mg/Kg OC and 284 mg/kg OL) for 3 months. A second group (B) of 10 patients consumed 40 ml/day of low OC/OL (82 mg/kg OC and 33 mg/kg OL) for 3 months. After a washout period of 9–12 months, a second intervention (DI2) only with High OC/OL-EVOO for 6 months was performed with 22 randomly selected patients (16 from the DI1 (8 from each group) and 6 new). Hematological, biochemical, and apoptotic markers were analyzed in the serum of the patients. In addition, cellular proliferation and apoptosis markers were studied in isolated proteins from peripheral blood mononuclear cells.

**Results:**

The results of the DI1 showed beneficial effects on hematological and apoptotic markers only with High OC/OL-EVOO. During the DI2, a decrease in the white blood cell and lymphocyte count was observed (p ≤0.05), comparing 3 months before the intervention and 6 months after it. After 3 and 6 months of DI2, an increase (p ≤0.05) was observed in the apoptotic markers ccK18 and Apo1-Fas, and also in the cell cycle negative regulator p21, and also a decrease in the antiapoptotic protein Survivin, and in the cellular proliferation marker Cyclin D.

**Conclusions:**

This is the first clinical trial with High OC/OL-EVOO that indicates that it could be a promising dietary feature for the improvement of CLL inducing the apoptosis of their cancer cells and improving the metabolism of the patients.

**Clinical Trial Registration:**

https://clinicaltrials.gov/ct2/show/NCT04215367, identifier: NCT04215367.

## Introduction

Daily intake of extra virgin olive oil (EVOO), which is the major component of the Mediterranean diet, may be partly responsible for the increased life expectancy of the Mediterranean people (1). More than 200 different chemical compounds have been detected in olive oil, namely, fatty acids, sterols, carotenoids, terpenoids, flavonoids, tocopherols, and olive phenols, which mainly correspond to tyrosol, hydroxytyrosol, oleocanthal, oleacein, ligstroside, and oleuropein aglycones. Olive phenols present a wide range of biological effects, namely, antioxidant, anti-inflammatory, anticancer, neuroprotective, and antidiabetic activities by the modulation of various molecular pathways (2, 3).

Chronic Lymphocytic Leukemia (CLL), the most commonly diagnosed adult leukemia in Western countries, is responsible for about 1 in 4 cases of all leukemias (4). It is characterized by the accumulation of monoclonal B cells in bone marrow, peripheral blood, lymphatic tissues, and spleen, while it is often asymptomatic and slow in its development. The standard clinical procedures to estimate prognosis are the clinical staging systems developed by Rai et al. and Binet et al. (5, 6). Patients at early stages of CLL do not require immediate therapy, offering the opportunity to perform interventions which could prevent or delay the disease progression. However, patients who appear to have symptoms have an approximate median survival range from 18 months to 6 years, depending on the clinical stage (7). The clinical course of the disease is extremely heterogeneous. Approximately one third of the patients never require treatment, but about the same percentage has to be treated early after diagnosis because of anemia, thrombocytopenia, lymphoadenopathies or splenomegaly and has a reduced life expectancy. The last one third of the patients start soon disease-related symptoms and require treatment at different times after diagnosis depending on external and genetic factors. There are not prognostic factors able to anticipate the disease progress (8).

CLL remains incurable in most of the patients regardless of the improvement in therapeutic schemes. Among the drugs which are giving an improvement in survival of the patients are purine analogues, monoclonal antibodies, Bruton tyrosine kinase inhibitors, phosphatidylinositol 3-kinase inhibitors, B-cell lymphoma 2 inhibitor, and CD20 monoclonal antibodies. Nonetheless there is a risk of resistance, a plethora of side effects, and a risk of drug toxicity. Other alternative therapeutic approaches include allogenic hematopoietic stem cell transplantations and chimeric antigen receptor T-cell therapy. Nevertheless, currently accessible therapies are partly effective in some patients (9). The big challenge is to be able to limit the progression of the disease through a dietary intervention, so that the patient will not need chemotherapy.

The level of apoptosis in hematopoiesis may provide information about the mechanism which is responsible for the regulation of the proliferation of the progenitor and stem cell. The dysfunction of apoptosis has been found to be associated with leukemia. Recent studies showed also that the increase in the lymphocytes of CLL patients may be due to the disturbed balance between the proliferation and apoptosis of CLL cells while the changes in apoptosis-related protein expression are recognized during all the stages of Binet in CLL (10). The apoptotic marker CD95 (Apo1-Fas) and its ligand, CD95L, have been considered as a death receptor/death ligand system that mediates apoptosis induction to maintain immune homeostasis (11). An increase in the expression of Apo1-Fas/CD95 apoptotic marker in myeloid progenitor cells in CLL patients and a corresponding decrease in the apoptotic BCL-2 inhibitor has also been described (12). Apo1-Fas also has the capacity to mediate apoptosis induction in cancer cells, generating by a caspase-specific processing of an 18-kilodalton fragment termed ccK18 (13). Oncogenes which activate Ras signaling pathway stimulate expression of ccK18 through transcription factors, and the elevated expression of ccK18 has been associated with an escape from the suppressive epigenetic mechanisms of chromatin condensation DNA methylation (14). RAS pathway is one of the pivotal cellular processes affected by novel mutations in CLL and is associated with adverse clinical features (15). Cytokeratins expression is not restricted to epithelial cancers, but can also be ‘‘aberrantly’’ detected in sarcomas, melanomas and lymphomas, anaplastic lymphomas, plasma cell neoplasms, T-cell lymphoma and can be detected in a small subset of otherwise characteristic B- and T-cell lymphomas as well (16).

Survivin, a protein inhibitor of apoptosis, inhibits caspases and blocks cell death. It is highly expressed in most cancers, including hematological, and is associated with a poor clinical outcome, high tumor grade cancers, lower disease survival and higher recurrence (17).

The relationship between diet and CLL progression has been studied in the past in patients with early stage CLL. In the MCC Spain study and also in the EPIC study, the association between the pro-inflammatory diet (measured by a higher DII score) and CLL was studied. The results of both studies suggest that chronic inflammation associated with diet might not play a strong role in CLL etiology. Nevertheless, using the MCC Spain data, an association between CLL and a high adherence to a Western dietary pattern was reported, independent with the proinflamatory diet (18). A recent case report described the effect of whole food plant-based (WFPB) diet intervention on the reduction of leukocytes and lymphocytes count during the intervention (19). Another dietary intervention with green tea extract Polyphenon E showed a sustained decline ≥20% in an absolute lymphocyte count. Although the Polyphenon E in this study was well tolerated, some patients present increased serum transaminase levels (20).

Among the olive oil secoiridoids, which contain elenolic acid or its derivatives in their molecular structures, oleocanthal has proven biological activity, namely, anticancer activity. Oleocanthal, the dialdehydic form of decarboxymethyl elenolic acid conjugated with *p*-(hydroxyphenyl)ethanol (*p*-HPEA-EDA), has been shown to induce cytotoxicity and apoptosis *in vitro* at a concentration equal or lower than 7.5 μM in human acute promyelocytic leukemia and in myeloma cells (21). It may also cause primary necrosis and cellular apoptosis by inducing lysosomal membrane permeability in different cancer cell lines (22). Although for several years oleocanthal was not possible to be detected in biological fluids, our recent studies demonstrated that oleocanthal could not be detected because it spontaneously reacts with aminoacids in plasma or in culture media, preferentially with glycine, creating oleoglycine. Disposition and pharmacokinetic studies performed in mice, as well as cell culture transport studies determined the ability of oleoglycine to cross physiological barriers such as the blood–brain barrier showing that it is potentially one of the active derivatives of oleocanthal circulating in the body (23).

Oleacein, the dialdehydic form of decarboxymethyl elenolic acid conjugated with 3,4-(dihydroxyphenyl)ethanol (3,4-DHPEA-EDA) has antioxidant, anti-inflammatory, and antimicrobial properties. Recent studies have found that oleacein has an antitumor activity against Multiple Myeloma (MM) cells through cell cycle arrest and apoptosis and by reducing clonogenicity without toxic effect on healthy cells (24). The same study has also shown an epigenetic effect of oleacein as demonstrated by the impairment of the MM acetylome, maybe *via* Sp1-dependent transcriptional inhibition of class I/II histone deacetylases.

Considering the strong apoptotic properties of oleocanthal and oleacein *in vitro* and *in vivo*, we wanted to investigate the effect of dietary intake of extra virgin olive oil rich in oleocanthal and the closely related oleacein (High OC/OL-EVOO) on hematological, metabolical, and cell progression markers and on disease progression in patients with Chronic Lymphocytic Leukemia. The aim was also to study the possible association of apoptosis in the mechanism of action of EVOO phenols in a patient with CLL. It should be made clear that the main purpose of the current pilot trial was to provide a proof of concept and to test if early stage CLL patients could adhere to and tolerate an intervention with high OC/OL EVOO and if this intervention could lead to any measurable positive effects, before starting a larger regular clinical trial.

## Materials and Methods

### Study Design and Participants

**Figure 1:**
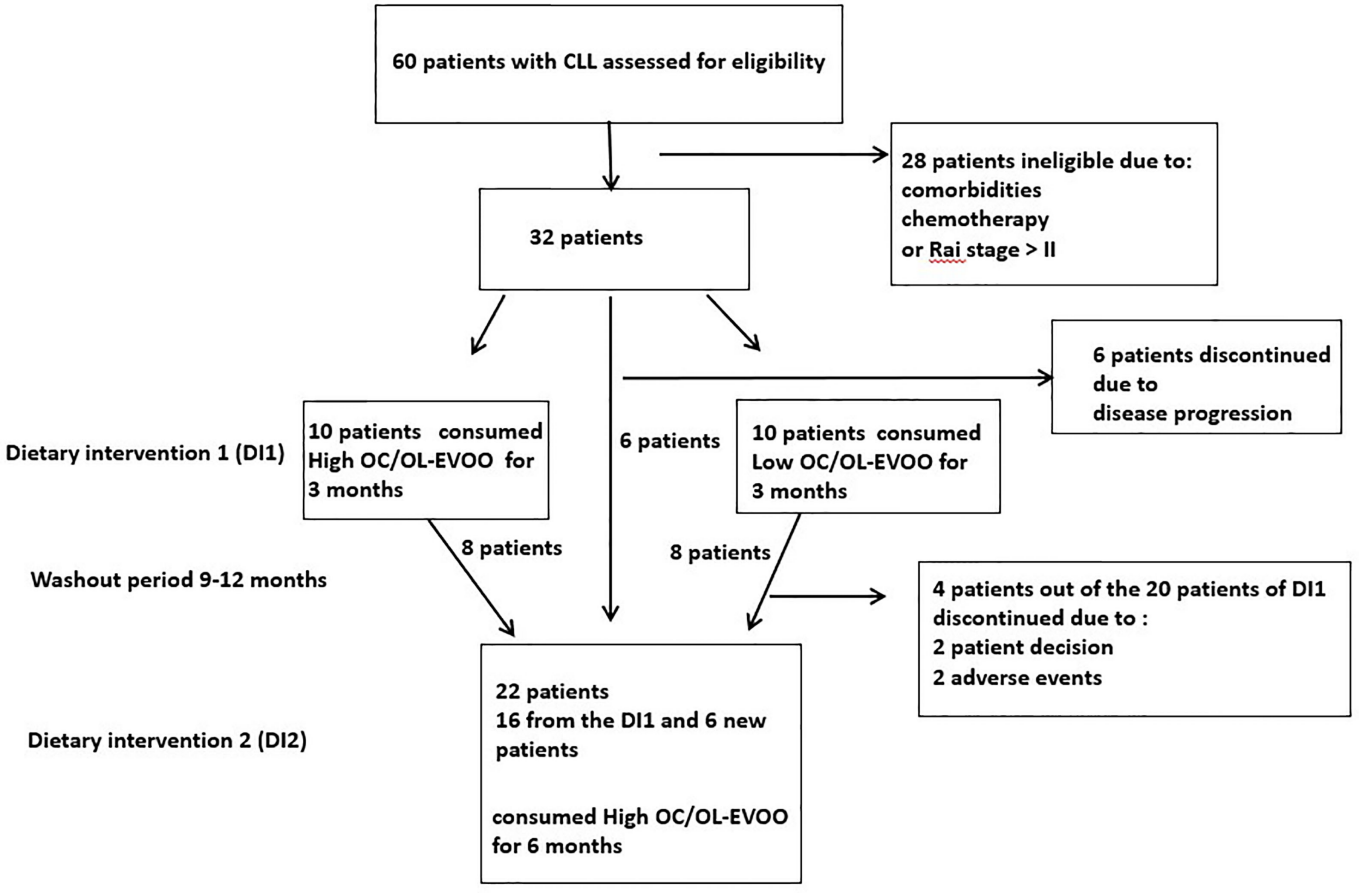
Trial profile: This figure displays the trial profile, including the total number of screened and randomized subjects, the group distribution in both dietary interventions, and an overview of reasons for withdrawal.

Patients from the hematologic clinic of the General Hospital of Laconia (Greece), were enrolled to participate in the dietary intervention (DI). CLL diagnosis was confirmed by standard criteria (6). Patients were untreated, in Rai stages 0 to II, did not meet criteria to initiate chemotherapy and had no neoplasmatic comorbidity. Exclusion criteria were any recorded neoplastic comorbidities, or insulin dependent diabetes, severe kidney disease, non-regulated thyroid disease, severe autoimmune disease, severe neurodegenerative disease and also chemotherapy treatment. Of the 60 patients with CLL in the hematology department, 32 had the required characteristics and the willingness to participate in the study. Of these 32 patients, 20 patients were randomly chosen for DI1. In order to avoid the biological effects of the DI1, a washout period [9 (minimum) to 12 (maximum) months after the finalization of the first intervention for each patient] was guarded. 22 patients were again randomly chosen to participate in DI2. Sixteen of them had participated in DI1 (8 from each group) and 6 were new patients (all of them belonging to the group of 32 initial patients). The trial profile with study design and patient selection information is depicted in **Figure 1**. All patients had high adherence to Mediterranean type diet which was assessed by 48 h recall and by MedDiet score (25).

### Procedures

#### Olive Oil Selection

Extra virgin olive oil (EVOO) samples obtained from olives (*Olea europaea* L.) harvested in 2016–2017 and 2018–2019 seasons were analyzed by qNMR to measure the phenol content as previously described (26). For the first dietary intervention (DI1) the following two types of oil, produced during the season 2016–2017, were chosen: EVOO low in oleocanthal and oleacein (Low OC/OL-EVOO) and EVOO high in oleocanthal and oleacein (High OC/OL-EVOO). The Low OC/OL-EVOO was a monovarietal oil of Koroneiki variety from ripe olives and contained: Oleocanthal 82 ± 5 mg/kg, oleacein 33 ± 3 mg/kg, tyrosol 250 ± 10 mg/kg, hydroxytyrosol 140 ± 9 mg/kg (D1 = 115 mg/kg, total phenolic content 505 mg/kg). The High OC/OL-EVOO was a monovarietal oil of Lianolia Kerkyras variety from Agios Matthaios, Corfu, Greece and contained: Oleocanthal 416 ± 7 mg/kg, oleacein 284 ± 10 mg/kg, oleuropein aglycon monoaldehyde 41 ± 3 mg/kg, ligstroside aglycon monoaldehyde 34 ± 3 mg/kg, oleokoronal 78 ± 4 mg/Kg, oleomissional 33 ± 3 mg/kg, tyrosol <10 mg/kg, hydroxytyrosol <10 mg/kg (D1 = 700 mg/kg). For the second dietary intervention (DI2), we chose an olive oil produced during the season 2018–2019, similar as possible with the High OC/OL-EVOO that was used in the DI1. Specifically, this High OC/OL-EVOO was a monovarietal oil of Lianolia Kerkyras variety from Agios Matthaios, Corfu, Greece and contained: Oleocanthal 415 ± 8 mg/kg, oleacein 267 ± 9 mg/kg, oleuropein aglycon monoaldehyde 50 ± 4 mg/kg, ligstroside aglycon monoaldehyde 49 ± 3 mg/kg, oleokoronal 76 ± 5 mg/kg, oleomissional 51 ± 4 mg/kg, tyrosol <10 mg/kg, hydroxytyrosol <10 mg/kg (D1 = 682 mg/kg). It should be mentioned that besides qNMR analysis, the content of free hydroxytyrosol and tyrosol was also measured by the standard international olive oil council (IOC) method for measurement of olive oil biophenols. All oils immediately after their analysis and during the study period were stored at 4°C to minimize possible alterations in chemical composition.

#### Dietary Interventions

##### Dietary intervention 1 (DI1)

**Figure 2:**
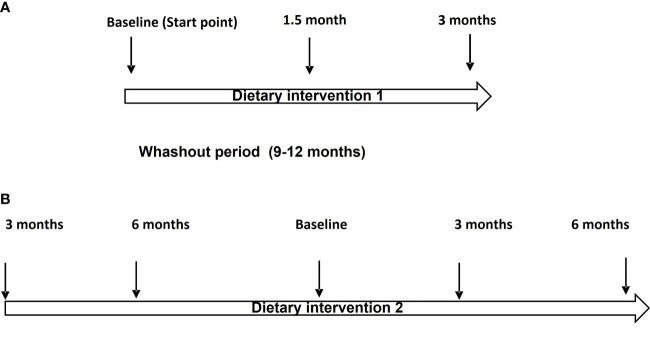
**(A)** Flow chart of the Dietary Intervention protocol (DI1). **(B)** Flow chart of the Dietary intervention protocol (DI2).

At the first stage (July 2017 to November 2017), 20 patients with CLL were blind randomly (block-randomization) separated in two groups (**Figure 1**). During this step of the dietary intervention (DI1) the participants consumed before their meals, 40 ml/day of either Low OC/OL-EVOO or High OC/OL-EVOO for 3 months to explore its effect on the hematological markers regarding white blood cells, lymphocytes, platelets, hematocrit and hemoglobin, and molecular apoptotic markers (Apo1-FAS, ccK18, and Survivin) at all the time points of the intervention (**Figure 2A**). The first group of 10 patients consumed Low OC/OL-EVOO and the second group of 10 patients consumed High OC/OL-EVOO. The amount of the consumed oil was chosen according to a previous study (27). Both EVOOs were provided in coded bottles of identical size, shape, and color. A detailed nutritional history was taken from each participant to ensure that their diet did not include olive oil rich in polyphenols (High OC/OL-EVOO) at the beginning of any dietary intervention. The enrolment, the random allocation, the assignment to intervention, the statistical analysis as well as the blinding were performed by different researcher of the team.

##### Dietary intervention 2 (DI2)

At the second stage, which was the main trial (https://clinicaltrials.gov/ct2/show/NCT04215367) (January 2019 to December 2019), twenty-two patients with CLL were randomly selected for DI2 only with High OC/OL-EVOO (40 mL/day before their meals) (**Figure 1**). The clinical record before the DI2, concerning the hematological and biochemical markers and also data concerning blood marrow biopsy and neoplastic markers were collected. Drug treatment or comorbidities were also recorded. During the 6 months period before the beginning of DI2, the patients did not consume High OC/OL-EVOO in their diet. The effect of the DI2 was investigated comparing the studied markers before (6 and 3 months), at the starting point (baseline), and during the DI2 (3 and 6 months) to evaluate possible sustained hematologic improvement according to the Rai et al. criteria, and the effect on biochemical–metabolic markers (**Figure 2B**). During the DI2, patients were advised to continue their regular diet and their adherence to the study protocol was assessed by telephone calls every two weeks.

### Measurements

#### Hematological and Biochemical Markers

Whole cell blood count regarding white blood cells, lymphocytes, platelets, hematocrit, and hemoglobin was measured at all the time points of the intervention by a hematological analyzer (Sysmex K-4500 (Toa Medical Electronics Co., Ltd., Kobe, Japan).

Biochemical markers measured in the present study were: Fasting glucose (mg/dl), lipidemic profile (cholesterol (mg/dl), Low Density Lipoprotein (LDL—mg/dl), High Density Lipoprotein (HDL—mg/dl), triglycerides (mg/dl)), markers of the liver function (Serum glutamic pyruvic transaminase (SGPT (U/L)), Serum glutamic oxaloacetic transaminase (SGOT(U/L)), lactate dehydrogenase (LDH(U/L)), alkaline phosphatase (ALP(U/L)), gamma-glutamyl transferase (γ-GT(U/L) and markers of kidney function (Urea(mg/dl) and creatinine (mg/dl). The serum analyses as a whole were conducted with the same procedure and at the same lab with the use of a biochemical analyzer (Olympus AU600. USA).

#### Molecular Apoptotic Markers

The apoptotic markers were investigated comparing the participants’ protein serum expression level in the peripheral blood sample by ELISA. The apoptotic proteins Apo1-Fas/CD95 (pg/ml) (Novus Biologicals, Colorado USA) and ccK18 (U/L) (MC30 ELISA used to detect caspase-cleaved CK18 produced during the early stages of apoptosis. VLVbio Hästholmsvägen, Sweden) and the antiapoptotic protein Survivin (pg/ml) (Origene—OriGene Technologies, Inc. USA) were measured according to the instructions of the supplier. Each experiment was performed in duplicate.

#### TUNEL Assay

The effect of the dietary intake of High OC/OL-EVOO on apoptosis was also investigated by TUNEL Assay in isolated peripheral blood mononuclear cells (PMBC) isolated from peripheral blood from the CLL patients during the dietary intervention. Approximately 20 ml of venous blood from the patients with CLL were obtained in a heparinized syringe (heparin, 90 IU/10 ml blood). The blood was overlaid on Ficoll–Paque (Histopaque 1077, Sigma, Aldrich Company Ltd, Dorset, UK) at a 2:1 ratio and centrifuged at 400*g* for 35 min at 20°C. PBMCs were collected from the interface and washed with PBS buffer (GIBCO Invitrogen Corporation, Carlsbad, CA, USA) three times to remove the plasma and the Ficoll, according to the manufacturer’s instructions. Isolated PBMC were transferred to the slides and fixed in 4% formaldehyde in PBS (pH 7.4) for 30 min at 4°C. Then the cells were washed twice in PBS and permeabilized in PBS containing 0.2% Triton x-100 for 30 min at room temperature. After washing with PBS, TUNEL was performed according to the manufacturer’s instructions (Biotium, Inc. Fremont, CA USA). DAPI staining at concentration 0.5 μM (Abcam UK) was performed incubating the slides for 30 min after the TUNEL assay.

#### Western Immunoblotting

From isolated PBMC, proteins were extracted with lysis buffer (50 mM of NaCl, 50 nM Tris, 1% Triton X 100, 1% Sodium deoxycholate and 0.1% SDS), protease inhibitors and 50 μg of protein which was loaded per lane. The proteins were separated by SDS–PAGE and transferred to nitrocellulose membrane. Membrane was blocked with 5% fat free dry milk in 1× TBS containing 0.05% Tween-20 for 1 h at room temperature. The blots were then probed with primary antibodies p21 (21 kDa) (1:1,000 dilution. Trans-duction Laboratories, Lexington, KY), Survivin (18 kDa) (1:500 dilution, Abcam, Cam-bridge UK), and cyclin D (36 kDa) (1:1,000, Santa Cruz Biotechnology, Santa Cruz, CA) overnight at 4°C and then incubated with horseradish peroxidase-labeled secondary antibody. Protein bands were detected by ECL. The visualization of β-actin (1:2,000) was used to ensure equal sample loading in each lane. Quantification of relative protein expression of the Western signals (complexed protein bands) was performed manually using the image analysis program, Image-J (IE 6.0, Microsoft Java).

#### Statistical Analysis

Analysis was performed using SPSS v.24 (SPSS Inc., Chicago, IL, USA) and the significance level was set at 0.05. Initially a descriptive analysis of the sample was performed in terms of age, sex, clinical, biochemical, and molecular markers. The markers were found to be skewed at p <0.05 significance level when checked by using the Kolmogorov–Smirnov test. Therefore, non-parametric Mann–Whitney U test was used to find out the differences between the intervention and the control group. The differences between the time intervals of the intervention were assessed with Friedman non‐parametric test followed by Dunn’s test for multiple comparison. Finally, the correlations of the molecular markers with the clinical parameters included in the study were checked with Spearman Rho. Data concerning the Western immunoblotting and TUNEL assay were compared using the unpaired two-tailed t-test.

## Results

### Dietary Intervention DI1

During the first step of the dietary intervention (DI1) the participants consumed Low OC/OL-EVOO or High OC/OL-EVOO for 3 months to explore its effect on the hematological markers (white blood cells, lymphocytes, platelets, hematocrit, and hemoglobin) and molecular apoptotic markers (Apo1-Fas, ccK18, and Survivin) at all the time points of the intervention. A statistically significant reduction in white blood cells was observed after three months in comparison with the baseline, only in the group of High OC/OL-EVOO. In contrast, the reduction observed in the Low OC/OL-EVOO group was not statistically significant. In addition, a statistically significant increase of serum molecular apoptotic markers (ccK18 and Apo1-Fas) and a decrease of the antiapoptotic protein Survivin were observed only in the group of High OC/OL-EVOO after three months. The apoptotic markers in the Low OC/OL-EVOO group did not show any significant changes. Detailed results are provided in **Table 1**.

**Table 1:** Differences in hematological and apoptotic markers of patients with CLL before and during the intervention DI1 with high OC/OL-EVOO (10 patients) and low OC/OL-EVOO (10 patients). *^a^*

Dietary intervention DI1 with High OC/OL-EVOO				
Whole blood count	Baseline a Median (min–max)	45 days b Median (min–max)	90 days c Median (min–max)	p-value (adj)
White Blood Cells (×10^3^/mm^3^)	16,900.00 (9,200–69,600)	12,950.00 (7,000–81,400)	11,700.00 (7,000–57,000)	**a–b 0.040** **a–c 0.000**
Lymphocytes (×10^3^/mm^3^)	10,800.00 (3,300–59,400)	7,925.00 (3,100–70,000)	7,900.00 (2,700–49,700)	0.138
Platelets (×10^3^/mm^3^)	214,000 (124,000–306,000)	221,500 (123,000–297,000)	201,000 (106,000–338,000)	0.432
Hematocrite (%)	42.70 (36.40–46.00)	41.95 (38.70–47.80)	41.65 (35.80–47.90)	0.740
Hemoglobin (g/dl)	14.35 (12.00–15.60)	13.75 (12.50–16.20)	13.90 (11.50–16)	0.934
Monocytes (×10^3^/mm^3^)	0.40 (0.10–2.10)	0.30 (0.10–2.20)	0.35 (0.20–1.60)	0.442
Neutrophils (×10^3^/mm^3^)	4.20 (1.70–8.00)	4.20 (1.40–8.20)	4.10 (1.80–6.92)	0.159
Eosinophils (×10^3^/mm^3^)	0.19 (0.06–0.54)	0.21 (0.06–1.30)	0.17 (0.06–0.44)	0.459
Basophils (×103/mm^3^)	0.04 (0.01–0.25)	0.04 (0.00–0.50)	0.04 (0.01–0.23)	0.669
ccK18 (U/L)	129.79 (47.78–389.32)	126.70 (68.12–585.58)	235.78 (76.51–998.00)	**a–c 0.046**
Apo1-Fas (U/L)	84.49 (59.29–528.88)	95.36 (66.01–611.81)	93.46 (54.86–805.41)	**a–c 0.030**
Survivin/API4 (pg/ml)	103.06 (86.62–133.13)	106.86 (85.31–181.89)	94.03 (32.00–160.00)	**b–c 0.024**
**Dietary intervention DI1 with Low OC/OL-EVOO**				
**Whole blood count**	**Baseline** **a** Median (min–max)	**45 days** **b** Median (min–max)	**90 days** **c** Median (min–max)	**p-value** **(adj)**
White Blood Cells (×10^3^/mm^3^)	22,100.00 (8,900–30,900)	21,433.33 (8,700–26,000)	20,400.00 (8,200–26,000)	0.131
Lymphocytes (×10^3^/mm^3^)	14,600.00 (3,300–24,400)	15,333.33 (3,300–21,700)	14,400.00 (2,800–21,700)	0.248
Platelets (×10^3^/mm^3^)	232,000 (140,000–289,000)	232,333.33 (134,000–295,000)	235,000 (125,000–321,000)	0.607
Hematocrite (%)	42.60 (37.40–45.70)	39.40 (12.70–47.80)	41.65 (39.60–46.80)	0.159
Hemoglobin (g/dl)	14.20 (12.50–15.60)	13.40 (12.80–16.20)	13.60 (12.70–15.60)	0.846
Monocytes (×10^3^/mm^3^)	0.60 (0.20–0.80)	0.40 (0.20–1.00)	0.50 (0.30–1.10)	0.211
Neutrophils (×10^3^/mm^3^)	5.20 (2.80–5.80)	4.20 (2.30–6.30)	4.50 (2.00–5.00)	0.143
Eosinophils (×10^3^/mm^3^)	0.17 (0.09–0.23)	0.23 (0.10–0.29)	0.20 (0.10–0.30)	0.196
Basophils (×103/mm^3^)	0.04 (0.02–0.08)	0.05 (0.04–0.06)	0.04 (0.02–0.09)	0.327
ccK18 (U/L)	117.91 (30.39–218.47)	112.40 (55.04–149.40)	107.71 (66.51–160.11)	0.882
Apo1-Fas (U/L)	91.69 (69.00–121.36)	87.62 (56.00–111.29)	91.32 (72.00–105.00)	1.000
Survivin/API4 (pg/ml)	103.55 (86.20–110.91)	110.87 (102.63–159.62)	114.13 (77.44–132.00)	0.368

a Analysis was carried using Friedman test and Dunn–Bonferroni post hoc tests.

Bold emphasize statistical significant values.

#### Subjects’ Characteristics for DI1

In the DI1 participated 20 patients, 10 in each group. In the intervention with high OC/OL EVOO participated 6 males (53%) and 4 females (47%) with mean age 71.10 ± 7.51. In the intervention with low OC/OL EVOO participated 4 males (47%) and 6 females (53%) with mean age 70 ± 7.96.

### Dietary Intervention DI2

At the second stage of the intervention (DI2), the participants consumed only High OC/OL-EVOO for six months. The effect of the DI2 was investigated comparing the hematological, biochemical, and apoptotic markers before (6 and 3 months), at the starting point (baseline), and during the DI2 (3 and 6 months) to evaluate possible hematologic improvement.

#### Subjects’ Characteristics for DI2

In the DI2, 22 patients participated with CLL: 12 males (54.5%) and 10 females (45.5%) with mean age 71.45 ± 7.53 years old.

#### Effects on Hematological Markers of DI2 With High OC/OL-EVOO

**Figure 3:**
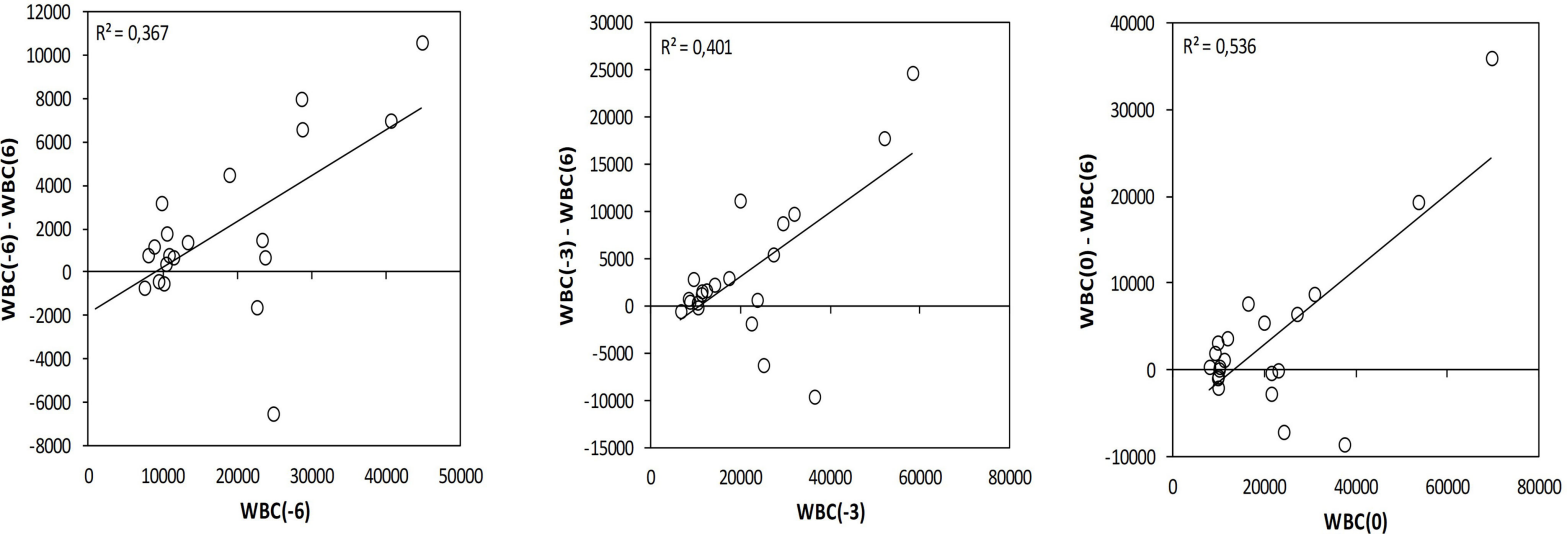
Variation on the white blood cells during the DI2 with High OC/OL**-**EVOO correlating the differences WBC_(−6)_ − WBC_(+6)_ with WBC(−6), WBC_(−3)_ − WBC_(+6)_ with WBC(−3) and WBC_(0)_ − WBC_(+6)_ with WBC_(0)_. Linear regression was performed and R^2^ is depicted.

To investigate the fluctuation of the studied hematological and biochemical markers, they were measured at 3 and 6 months before the intervention (**Table 2**). During the dietary intervention DI2, a statistically significant decrease in the white blood cells (WBC) and in the lymphocytes count was observed comparing the values 3 months before the intervention and 6 months after the beginning (**Table 2**). The WBC showed a greater decrease in patients who initially had higher WBC. WBC_(−6)_ (6 months before) showed a statistically significant correlation with WBC_(−6)_ − WBC_(+6)_ difference (R = 0.367, p = 0.000) and WBC_(0)_ (at baseline) showed a statistically significant correlation with WBC_(0)_ − WBC_(+6)_ difference (R = 0.536, p = 0.000) (**Figure 3**).

**Table 2:** Differences in hematological markers of patients with CLL (N = 22) before and during the DI2 with High OC/OL**-**EVOO. *^a^*

Whole blood count	6 months before a Median (min–max)	3 months before b Median (min–max)	Baseline c Median (min–max)	3 months after d Median (min–max)	6 months after e Median (min–max)	p-value (adj)
White Blood Cells (×10^3^/mm^3^)	13,300.00 (7,500–44,800)	18,650.00 (6,700.00–58,300.00)	16,900.00 (8,000.00–69,600.00)	13,100.00 (7,000.00–57,000.00)	11,900.00 (6,600.00–46,000.00)	**b–e 0.004**
Lymphocytes (×10^3^/mm^3^)	10,500.00 (3,000–43,200)	11,650.00 (3,300.0–50,600.0)	10,800.00 (3,300.00–59,400.00)	8,350.00 (2,700.00–49,700.00)	8,000.00 (2,500.00–39,400.00)	**b–e 0.000**
Platelets (×10^3^/mm^3^)	209,000 (124,000–277,000)	202,000 (152,000–309,000)	205,000 (124,000–306,000)	201,000.00 (106,000–338,000)	197,000.00 (101,000–321,000)	0.944
Hematocrite (%)	42.50 (38.50–49.80)	42.65 (36.10–49.80)	42.70 (36.40–48.40)	42.15 (35.80–49.50)	41.50 (36.00–49.20)	0.093
Hemoglobin (g/dl)	14.30 (12.80–16.30)	14.15 (12.10–16.30)	14.35 (12.00–15.80)	14.10 (11.50–16.00)	13.90 (11.70–16.50)	0.273
Monocytes (×10^3^/mm^3^)	0.30 (0.10–1.00)	0.55 (0.10–2.00)	0.40 (0.10–2.10)	0.35 (0.20–1.60)	0.30 (0.10–1.20)	0.176
Neutrophils (×10^3^/mm^3^)	3.80 (1.70–7.50)	4.95 (2.20–8.90)	4.20 (1.70–8.00)	4.10 (1.80–6.92)	3.80 (1.60–6.80)	**b–d 0.006** **b–e 0.003**
Eosinophils (×10^3^/mm^3^)	0.19 (0.05–0.61)	0.21 (0.06–0.64)	0.19 (0.06–0.54)	0.17 (0.06–0.44)	0.2 (0.07–0.60)	0.524
Basophils (×103/mm^3^)	0.03 (0.00–0.36)	0.08 (0.00–1.26)	0.04 (0.01–0.25)	0.04 (0.01–0.23)	0.02 (0.00–0.12)	**b–e 0.037**

a Analysis was carried using Friedman test and Dunn–Bonferroni post hoc tests.

Bold emphasize statistical significant values.

#### Effects on Biochemical Markers of DI2 With High OC/OL-EVOO

Variations in the biochemical markers during the DI2 are depicted in **Table 3**. A significant decrease regarding the glucose level was observed during the dietary interventions as shown in **Table 3**. There was no significant effect on CLL patients’ metabolic indexes concerning lipidemic profile and renal function markers during the dietary intervention. Regarding the hepatic function markers, an increase in alkaline phosphatase during the first 3 months of the dietary intervention (p = 0.024) was observed, which was followed by a reduction, reaching lower levels than the baseline (p = 0.039). Nevertheless, alkaline phosphatase level was within the normal limits (**Table 3**).

**Table 3:** Differences in biochemical markers of patients with CLL (N = 22) before and during the DI2 with High OC/OL**-**EVOO. *^a^*

Biochemical Marker	6 months before a Median (min–max)	3 months before b Median (min–max)	Baseline c Median (min–max)	3 months after d Median (min–max)	6 months after e Median (min-max)	p-value (adj)
Glucose (mg/dl)	103.00 (86.00–123.00)	103.50 (86.00–138.00))	104.00 (62.00–132.00)	100.00 (58.00–125.00)	98.00 (74.00–120.00)	**b–e 0.039** **c–e 0.018**
Urea (mg/dl)	40.00 (25.00–68.00)	41.50 (30.00–83.00)	43.50 (30.00–85.00)	40.00 (32.00–76.00)	40.00 (30.00–60.00)	0.154
Uric Acid (mg/dl)	5.40 (3.00–830)	5.40 (2.80–7.10)	5.50 (3.20–53.00)	5.53 (3.10–8.60)	5.20 (2.80–8.50)	0.578
Creatinin (mg/dl)	1.00 (0.70–1.20)	0.94 (0.60–1.40)	0.92 (0.60–1.40)	0.89 (0.70–1.40)	0.90 (.60–1.45)	0.061
SGPT (U/L)	19.00 (11.00–66.00)	19.50 (8.00–46.00)	18.50 (8.00–34.00)	21.50 (10.00–74.00)	15.00 (8.00–35.00)	0.083
SGOT (U/L)	24.00 (14.00–51.00)	25.00 (13.00–54.00)	26.40 (13.00–40.00)	22.67 (11.00–47.00)	22.00 (12.00–38.00)	0.329
LDH (IU/L)	201.00 (104.00–616.00)	217.00 (104.00–576.00)	213.00 (128.00–596.00)	234.50 (164.00–445.00)	227.50 (150.00–641.00)	0.058
γGT (U/L)	15.00 (10.00–38.00)	16.00 (9.00–45.00)	15.00 (9.00–39.00)	16.33 (10.00–37.00)	15.00 (7.00–39.00)	0.149
ALP (U/L)	73.00 (47.00–90.00)	71.00 (38.00–240.00)	69.00 (46.00–220.00)	81.00 (42.00–198.00)	75.00 (34.00–198.00)	**c–d 0.024** **c–e 0.039**
***Lipidemic Profile***						
Total cholesterol (mg/dl)	197.00 (137.00–277.00)	196.50 (137.00–255.00)	185.50 (140.00–305.00)	185.50 (125.00–300.00)	190.00 (125.00–286.00)	0.274
Triglycerides (mg/dl)	108.50 (46.00–250.00)	114.00 (49.00–256.00)	115.50 (63.00–299.00)	109.00 (45.00–298.00)	97.00 (67.00–280.00)	0.955
HDL cholesterol (mg/dl)	60.50 (34.00–92.00)	59.50 (36.00–108.00)	58.67 (33.00–99.00)	56.67 (16.00–98.00)	57.00 (35.00–82.00)	0.103
LDL cholesterol (mg/dl)	114.50 (72.00–185.00)	113.00 (70.00–182.00)	101.00 (68.00–212.00)	111.00 (73.00–190.00)	110.00 (68.00–200.00)	0.428

a Analysis was carried using Friedman test and Dunn–Bonferroni post hoc tests.

Bold emphasize statistical significant values.

#### Effects on Apoptotic Markers of DI2 With High OC/OL-EVOO

**Figure 4:**
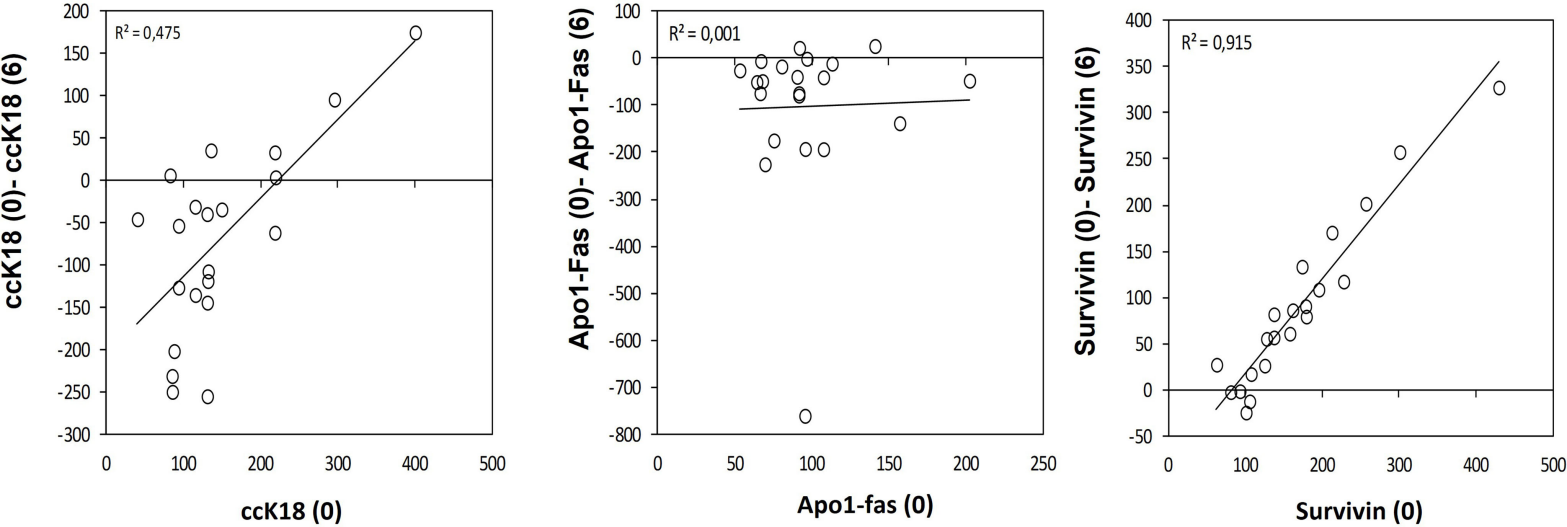
Variation on the apoptotic marker ccK18 **(A)**, Apo1-Fas **(B)** and antiapoptotic Survivin **(C)** during the dietary intervention DI2 with High OC/OL**-**EVOO. Linear regression was performed and R^2^ is depicted.

**Figure 5:**
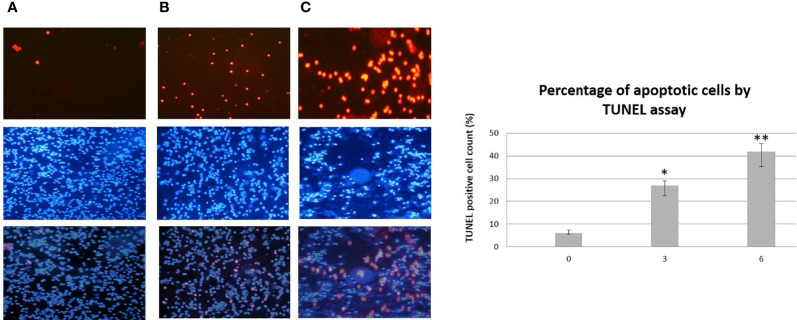
TUNEL Assay (top) performed on isolated PMBC from patients with CLL during the dietary intervention DI2 with High OC/OL**-**EVOO. **(A)** Baseline. **(B)** 3 months of dietary intervention. **(C)** 6 months of dietary intervention. DAPI staining (middle) and overlay (bottom) are also illustrated. The data in the histograms are the mean ± SD of 22 different experiment subjects. Data were compared using the unpaired two-tailed t-test. Significant difference between the time points of the dietary intervention at p <0.05 (*0–3, **3–6 and 0–6) is indicated. For each subject each experiment was performed twice.

**Figure 6:**
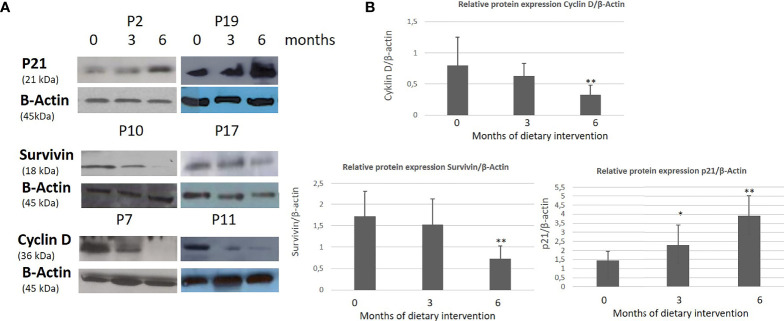
Effect of High OC/OL**-**EVOO on cyclin D, Survivin and p21 protein expression in PMBC cells of patients with CLL during DI2. The protein expression level was measured by Western blot analysis at baseline, at 3 and 6 months after dietary intervention in equal amount of cell lysate (40 lg/lane) from isolated PMBC cells of each participant. Cell lysates was subjected to electrophoresis and analyzed by Western blot. **(A)** Representative blots shown for each studied protein for selected patients (P2, 7, 10, 11, 17, 19). **(B)** The densitometry measurements are depicted in histograms. The data in the histograms are the mean ± SD of 22 different experiment subjects. The protein expression levels are normalized according to the measurement of β-actin in a semi quantitative analysis. Quantification of relative protein expression of the Western signals (complexed protein bands) was performed manually using the image analysis program, Image-J (IE 6.0, Microsoft Java). Data were compared using the unpaired two-tailed t-test. Significant difference between the time points of the dietary intervention at p <0.05 (*0–3, **3–6 and 0–6) is indicated.

A statistically significant increase in all patients with CLL during the dietary intervention was observed (p ≤0.05) in ccK18 and Apo1-Fas and also a statistically significant decrease in Survivin after 3 months which remained after 6 months (**Table 4**). Regarding the protein expression of the ccK18 in patients during the dietary intervention, it was observed that those patients who initially had a high ccK18 level also showed a higher increase in ccK18 after the intervention compared to those with lower initial ccK18 (R = 0.475, p = 0.001) (**Figure 4**). A negative correlation between the WBC at the end of the dietary intervention with the initial ccK18 and with the fluctuation of the protein expression of ccK18 (final − initial) (R = −0.477 p = 0.029) was also observed. Also, subjects with a decrease in Survivin and with a high initial Survivin showed greater reduction in Survivin compared to those with lower initial Survivin (R = 0.915, p = 0.044) (**Figure 4**). Regarding the TUNEL Assay performed in isolated PMBC of the patients with CLL, a statistically significant increase of the percentage (40%) of apoptotic cells was observed after the dietary intervention DI2 (p ≤0.01) (**Figure 5**). Concerning the protein analysis of apoptotic markers, the Western blot analysis revealed that the dietary intervention with High OC/OL-EVOO resulted in a decrease of the protein expression of Survivin and the positive cell cycle regulator protein cyclin D, and an increase of the protein expression of p21, a negative regulator of the cell cycle (**Figure 6**).

**Table 4:** Differences in apoptosis markers of patients with CLL (N = 22) during the DI2 with High OC/OL**-**EVOO. *^a^*

Apoptotic Markers	Baseline a Median (min–max)	3 months b Median (min–max)	6 months c Median (min–max)	p-value (adj)
ccK18 (U/L)	130.78 (40.39–400.45)	128.56 (46.94–585.58)	220.00 (76.50–385.50)	**a–c 0.013** **b–c 0.013**
Apo1-Fas (U/L)	91.84 (53.33–202.31)	95.36 (66.01–611.81)	140.87 (70.00–855.41)	**a–c 0.000** **b–c 0.025**
Survivin/API4 (pg/ml)	159.70 (62.65–429.50)	107.64 (62.13–256.58)	86.60 (35.00–125.00)	**a–c 0.000** **b–c 0.002**

a Analysis was carried using Friedman test and Dunn–Bonferroni post hoc tests.

Bold emphasize statistical significant values.

#### Correlation Analysis of the DI2 With High OC/OL-EVOO

Concerning the apoptotic markers, a negative correlation of the WBC at the end of the dietary intervention with the initial ccK18 (R = −0.502, p = 0.050) and with the fluctuation of the protein expression of ccK18 (final − initial) (R = −0.477 p = 0.029) was observed. Also, a positive correlation between the WBC at the end of the dietary intervention and the final p21 protein level expression (R = 0.352 p = 0.005) was found.

A negative correlation between the variation of the protein expression of ccK18 during the intervention and the initial cholesterol (R = −0.451 p ≤0.01) was also observed. Negative was the correlation between SGOT and LDH at the beginning of the dietary intervention with the variation of the protein expression of ccK18 during the intervention (R = −0.452 p ≤0.05 and R = −0.537 p ≤0.05, respectively). It was also observed a positive correlation between SGOT and LDH at the beginning of the dietary intervention with the final WBC (R = 0.456 p ≤0.05 and R = 0.492 p ≤0.05, respectively).

Concerning the biochemical markers, a positive correlation between glucose and WBC at the end of the dietary intervention (R = 0.454, p ≤0.05) was observed. A positive correlation between triglycerides and WBC at the end of the DI2 (R = 0.559, p ≤0.01) was found.

## Discussion

One of our main targets was to investigate the hypothesis that the biological activity of olive oil against cancer is related to its content in specific phenolic ingredients and especially in oleocanthal and oleacein (index D1), as we had recently shown for the inhibition of platelet aggregation (27) and for the improvement of cognitive function in people with mild cognitive impairment (2).

Natural compounds, which may act preventing the onset and progression of primary cancer, have been found in plant-based products such as vegetables, fruits, herbs, and fermented products. Polyphenols from EVOO have been studied demonstrating beneficial effects in *in vivo* and *in vitro* studies against several types of cancer (3). Previous nutrition-related studies and nutritional interventions have shown that a plant-based nutrition and a Mediterranean type diet could be beneficial to prevent CLL progression. Also, during intervention with Polyphenon E from green tea, a reduction in the absolute lymphocyte count had been observed (18–20).

The present study attempted to investigate the effect of nutritional intervention with High OC/OL-EVOO on hematological, biochemical, and metabolic markers and also molecular markers associated with apoptosis and cell survival, in patients with chronic lymphocytic leukemia in Rai stages 0–II who did not follow any treatment. Since this study was a pilot and not a regular clinical trial, we were not targeting to offer a proof of efficacy but mainly of a proof that High OC/OL-EVOO can be tolerated by CLL patients and can have measurable effects on them, making worthy a future investigation through a larger clinical trial.

Data that support the ability of oleocanthal to be metabolized in the body are found in recent studies. Specifically, the intestinal absorption of oleocanthal in rats has been reported, which is extensively metabolized by phase I and II metabolizing enzymes (28). However, another study of our research group has demonstrated that oleocanthal in plasma is mainly transformed to derivates such as oleoglycine able to cross physiological barriers (23).

In the present survey a statistically significant reduction of WBC and lymphocytes was observed after the dietary intervention while hematocrit and platelet levels were stable in patients with CLL. *In vivo* surveys have linked EVOO consumption with reduced incidence of cancer (29). An *in vivo* study in nude mouse xenograft reported prevention of breast cancer tumor recurrence associated with downregulation of MET and HER2 receptors and suppression of receptor activation after daily oral treatment with 10 mg/kg oleocanthal (30). Several *in vitro* and *in vivo* studies have described that oleocanthal is toxic to many cancer cell lines and causes loss of cell viability without killing healthy cells (31). Olive oils with high oleocanthal content could effectively destroy a variety of cancer cells in a manner similar to purified oleocanthal. The cytotoxic effects can be achieved due to the ability of oleocanthal to induce apoptosis, lysosome membrane permeabilization or necrosis preferentially in cancer cells (22).

CLL remain incurable in most patients. Recent therapeutic strategies against CLL aim to block important cellular signal transduction pathways involved in pivotal processes including proliferation, differentiation, apoptosis, and cell migration. However, serious complications are observed in this type of therapy as they can produce toxicity especially in elderly patients such as those participating in our study. The clinical challenge is to be able to block the progress of the disease in its early stages so that there is no need for the patient to start any chemotherapeutic schedule (8, 9, 32).

The current study indicates the possible ability of the High OC/OL-EVOO to induce apoptosis and cell cycle arrest in patients with early stage CLL. During the dietary intervention (both DI1 and DI2) an increase in the serum protein expression of the apoptotic markers ccK18 and Apo1-Fas and a decrease in the serum protein expression of the antiapoptotic protein Survivin was observed. Moreover, a negative correlation of the WBC at the end of the dietary intervention with the fluctuation of the protein expression of the apoptotic marker ccK18 (final − initial) was observed.

Evading the apoptotic process is one of the characteristics of cancer which represent a pivotal mechanism in clinical resistance to therapies. This is particularly a fact for chronic lymphocytic leukemia (CLL), which is clearly characterized by impaired apoptosis. The development of therapeutic support strategies that target apoptosis in CLL is therefore a very important issue (33).

In the current survey, the protein expression analysis of the isolated PMBC from the patients, by western immunoblotting, showed a decrease in the protein expression of Survivin and cyclin D and an increase of the p21^WAF1^ protein expression, indicating a negative cell cycle effect of the DI2 with High OC/OL-EVOO. Additionally, an increase of the apoptotic rates of the isolated PMBC during the DI2 was registered by TUNEL assay. Furthermore, a positive correlation of the WBC at the end of the dietary intervention was observed with the final p21 protein level expression.

CLL is characterized by low proliferative activity characterized by the accumulation of clonal B lymphocytes arrested in G0/G1 phase of the cell cycle. Previous research has focused on the defective apoptosis of the malignant cells that seems to play an important role in disease progression and chemotherapy resistance. Overexpression of cyclin D2 mRNA has been described in B-CLL cells. Cyclins D-type regulates early G1 progression and are thought to be growth factor sensors linking extracellular signals to the cell cycle (34). Moreover, the cyclin-dependent kinase inhibitor p21^WAF1^ is a regulator of cellular processes such as DNA replication, cell cycle arrest, and apoptosis. There is evidence that disruption of p53/p21 is an important prognostic marker of CLL (35). Also, the increase of p21 expression has been studied as probable therapeutic target in CLL the last years (36).

Oleocanthal has already been shown to induce cytotoxicity and apoptosis *in vitro* in human acute promyelocytic leukemia HL-60 cells, myeloma ARH-77 cells and murine myeloma MOPC-31C, to cause G1 arrest in ARH-77 cells and to promote their apoptosis by the activation of caspase-9/-3. It has been demonstrated that oleocanthal could induce apoptosis through the suppression of Akt/RANKL (receptor activator of nuclear factor B ligand) which could lead to p38 MAPK signaling a caspase-9 activation or through the induction of cleaved caspase-3 and cleaved PARP (9). A recent research showed that the described cytotoxic effects of EVOOs are related to their content in oleocanthal (22). To further support the role of oleocanthal in the current trial, we studied the apoptotic effect of pure oleocanthal in isolated PMBC cells from one patient with CLL that participated in the trial. The clear apoptotic activity observed at concentration of 25 μM is presented at the supplementary material.

Oleacein also plays an important role through its ability to trigger a tumor suppressive miRNA network likely contributing to its cytotoxicity against multiple myeloma cells. Also, oleacein acts *via* epigenetic mechanisms inducing apoptosis (24).

Although CLL cells undergo spontaneous apoptosis as mentioned, a gradual increase in the size of the CLL clone resulting from newly born lymphocytes is observed and the cells’ metabolic program is reprogramed to adjust for their increased proliferation rate. Changes include adaptation to carbohydrate and lipid metabolism. There is evidence for altered metabolism, increased mitochondrial activity and formation of reactive oxygen species (ROS) in CLL cells, and for this reason possible therapeutic strategies oriented to amend metabolism pathways in patient with CLL have pivotal importance (37).

Concerning the correlation between oleocanthal and the biochemical profile of the studied patients, the results of the present study demonstrated that the DI2 had a beneficial effect on glucose metabolism. It is already known from previous studies that leukemic cells could induce a diabetic state in patients and also desensitize normal tissues to glucose; so neoplastic leukemia cells could deprive from normal cells the glycolytic capacity and at the same time increase the availability of glucose to drive their own growth. Leukemia cells could induce insulin resistance *via* multiple mechanisms (38). A previous study has showed that daily consumption of polyphenol-rich EVOO might improve metabolic control, including fasting glucose and HbA1c probably through the decrease of circulating inflammatory adipokines profile in overweight T2D patients (39).

Concerning the effect of the DI2 on the lipidemic profile, correlation analysis showed a positive correlation between triglycerides and WBC at the end of the DI2 as well as a negative correlation between the variation of the protein expression of the apoptotic marker ccK18 during the intervention and the initial cholesterol. *In vitro* studies have found that cholesterol, and in particular its levels in the outer membrane of mitochondria, appear to have the ability to alter the solubility and thus inhibit the activation of the Bax protein, while they increase the resistance to stress-induced apoptosis. This also appears to be one of the mechanisms through which statins exert their pre-apoptotic effect (40). Other research studies have reported that the stage and activity of CLL disease may be affected by the levels of the total cholesterol, HDL and LDL levels (41).

Therefore, the maintaining of normal levels of lipids during the intervention could be considered as a beneficial result of High OC/OL-EVOO action, probably also influencing the mechanisms of apoptosis or survival of cancer cells.

Regarding the hepatic function markers, the present survey observed a positive correlation between the final WBC and SGOT and LDH at the beginning of the DI2 as well as a negative correlation between SGOT and LDH at the beginning of the DI2 with the variation of the protein expression of ccK18 during the intervention. A recent study determined that patients with liver dysfunction may have shorter overall survival compared to CLL patients with normal liver function (42). LDH is a ubiquitous enzyme with an important role in tumor behavior. LDH plays a crucial role in the Warburg effect, which is the phenomenon that cancer cells could switch from an aerobic to a predominantly anaerobic mechanism, a common feature of malignant cells—important for their tumorigenic potential (43). Despite the correlation of the baseline liver markers with the final WBC, their levels remained at normal levels with a decrease during the intervention. There are indications about the beneficial role of the High OC/OL-EVOO, regarding the maintenance of the hepatic function and its effect on the CLL progression.

To summarize, after taking the above-described data into consideration it is possible to consider the ability of oleocanthal and oleacein to increase apoptosis and reduce the survival of lymphocytes. This condition may happen even after a short time of intervention, in more deteriorated CLL clinical conditions.

The present study had some limitations, especially regarding the number of CLL patients and the duration of the intervention. We cannot exclude the possibility that the observed effects on CLL are also related to other unknown minor constituents of olive oil that were not measured during this study. It is also important to mention that according to recent studies there are more current and accurate techniques to use for molecular monitoring of CLL patients’ outcome (for example regarding to measurement of CLL clone size and minimally detectable disease) which could be used in future studies in order to achieve more detailed information about the EVOO-OC/OL action on early CLL progress.

However, this is the first pilot dietary intervention study, in which there are strong indications of the beneficial effect of the oleocanthal and oleacein in the progression of CLL.

## Conclusions

The complex mechanisms regarding the progression of CLL disease appear to benefit from the anticancer properties of oleocanthal and/or oleacein. Specifically, it is the first time that we were able to show that oral administration of a daily dose of 25 mg oleocanthal and oleacein through the consumption of 40 ml of EVOO could be beneficial for CLL patients, inducing the apoptosis of their cancer cells and improving the metabolism of the patients.

Further investigation of both mechanisms of negative CLL progression and effect of oleocanthal and/or oleacein, in long term dietary intervention studies with greater number of participants is necessary. In this manner, it could be possible to clarify the precise role of oleocanthal and the capacity of such dietary interventions, to increase life expectancy and to stabilize the development and progression of neoplastic blood diseases such as CLL.

The results of this pilot study should not be used as a proof of efficacy but as a proof that the study can and is worthy to be expanded to a multi-center larger trial.

## Data Availability Statement

The original contributions presented in the study are included in the article/**Supplementary Material**. Further inquiries can be directed to the corresponding author.

## Ethics Statement

The studies involving human participants were reviewed and approved by the Ethic and Scientific committee of the General Hospital of Laconia (23158/29-06-2017 and 44917/13-12-2018) and the Research & Ethics Committee of the University of Peloponnese (14/5/2020). The patients/participants provided their written informed consent to participate in this study.

## Author Contributions

Conceptualization, AG, IK, EM, and PM. Methodology, AG, IK, AI, and TN. Formal analysis, AI and TN. Investigation, AG, IK, GK, MK, and EM. Resources, EM. Data curation, ID. Writing—Original draft preparation, AG. Writing—Review and editing, EM and PM. Supervision, PM. Project administration, PM. All authors contributed to the article and approved the submitted version.

## Conflict of Interest

The authors declare that the research was conducted in the absence of any commercial or financial relationships that could be construed as a potential conflict of interest.

## Publisher’s Note

All claims expressed in this article are solely those of the authors and do not necessarily represent those of their affiliated organizations, or those of the publisher, the editors and the reviewers. Any product that may be evaluated in this article, or claim that may be made by its manufacturer, is not guaranteed or endorsed by the publisher.
