# Supplementary material for: The Effect of Dietary Intervention With High-Oleocanthal and Oleacein Olive Oil in Patients With Early-Stage Chronic Lymphocytic Leukemia: A Pilot Randomized Trial
Source: Front Oncol. 2022 Jan 21;11:810249. doi: 10.3389/fonc.2021.810249 (PMC8814521; doi:10.3389/fonc.2021.810249)
Supplement: Supplementary file 1 [file DataSheet_1.docx]

**SUPPLEMENTARY MATERIAL**

**Study of the apoptotic effect of pure oleocanthal on PBMC cells isolated from one patient that participated in the trial**

Methods

The PMBC cells were isolated according to the manufacturer`s instructions (Histopaque 1077, Sigma, Aldrich Company Ltd, Dorset, UK). Using the Viability/Cytotoxicity Assay (Biotium Fremont, CA USA) non cytotoxic dose of oleocanthal was founded (25μM).

PMBC cells from CLL patient was treated for 48 hr with 25μM of oleocanthal or 0 μM of oleocanthal (as a negative marker). After twice washes with PBS cells were stained for Tunnel assay and immunofluorescence.

Tunnel assay was performed according to the manufacturer’s instructions (Biotium, Inc. Fremont, CA USA).

The cellular expression of an apoptotic marker Cathepsin E was studied with immunofluorescence. For the first part of the experiments, the cells were analyzed at the basal state or after induction with 25 μΜ oleocanthal, the cells were washed twice with phosphate-buffered saline (PBS) and immunofluorescence was performed. The primary antibody was applied at an appropriate dilution, time and temperature (cathepsin 1:100, overnight at 4 °C), (Acris, USA). One-hour incubation period with the secondary antibodies was performed [fluorescein isothiocyanate (FITC) goat anti-rabbit immunoglobulin G (IgG)-FITC, sc-2012, 1:100, Santa Cruz Biotechnology, Inc., Dallas, TX, USA]. The cells were then wash the cells in PBS as needed and stained with 300 nM DAPI stain solution for 1–5 minutes, protected from light. After washing the cells were imaged

The images were acquired using a Nikon Eclipse TE 2000-U, Phihong Enterprise, Taiwan. The immunofluorescence data were quantified with the use of the computer program “ImageJ, National Institutes of Health, University of Wisconsin, WI, USA”.

**Results**

**At basal state**

DAPI Cathepsin


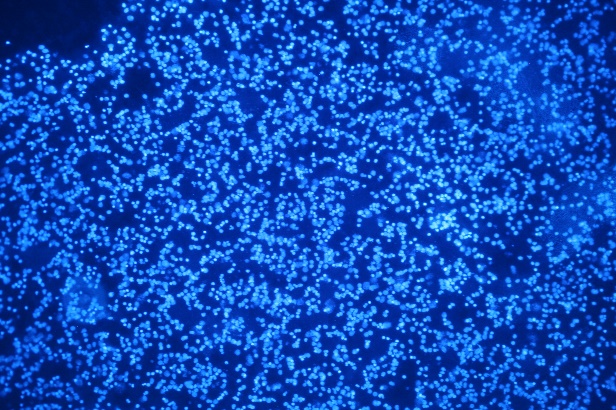

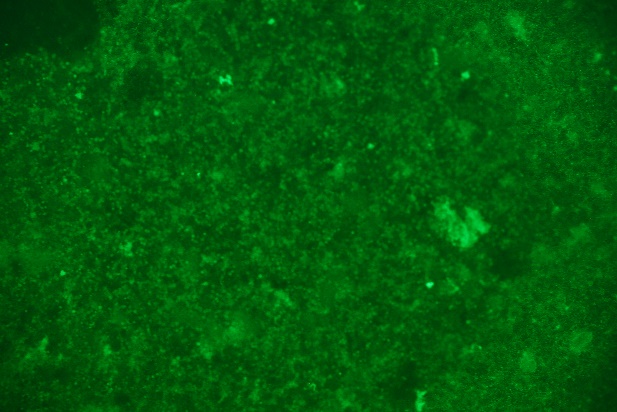


**After induction with 25 μΜ oleocanthal**

DAPI Cathepsin


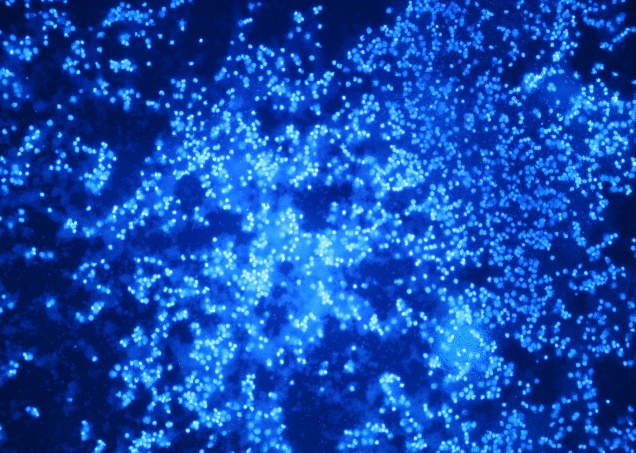

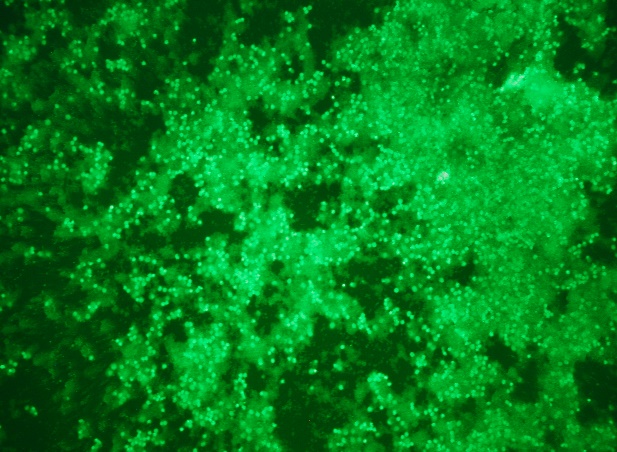


TUNEL Assay

At basal state After induction with 25 μΜ oleocanthal


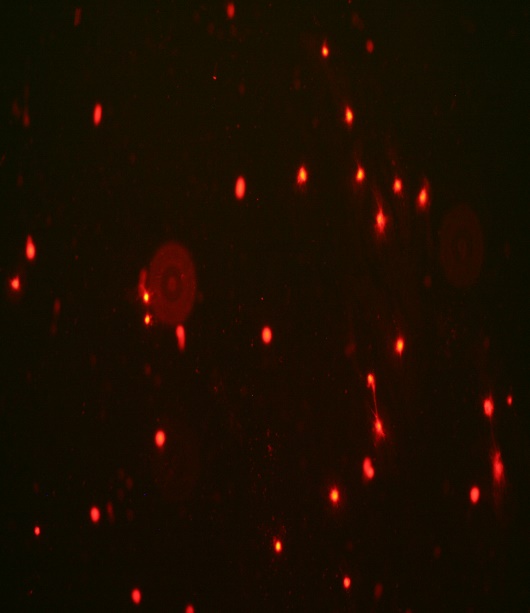

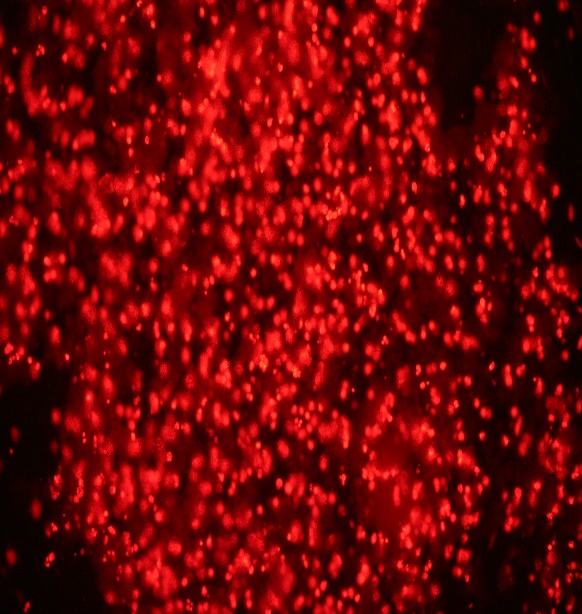


**Table S1.** Differences in hematological markers of patients with CLL (N=22) before and during the DI2 with High OC/OL**-**EVOO (M: male, F: female)

| **Whole blood count** | **Sex** | **6 months before**  **a**  Median  (min-max) | **3 months before**  **b**  Median  (min-max) | **Baseline**  **c**  Median  (min-max) | **3 months after**  **d**  Median  (min-max) | **6 months after**  **e**  Median  (min-max) | **p-value (adj)** |
| --- | --- | --- | --- | --- | --- | --- | --- |
| White Blood  Cells  (x10^3^/mm^3^) | M | 13300.00  (8000-44800) | 18650.00  (6700-52000) | 16900.00  (8000-53600) | 13050.00  (7100-46100) | 11900.00  (7200-46000) | 0.199 |
|  | F | 17000.00  (7500-40600) | 17400.00  (8700-58300) | 16700.00  (9800-69600) | 16350.00  (7000-57000) | 15650.00  (6600-33600) | **b-e 0.019** |
| Lymphocytes  (x 10^3^/mm^3^) | M | 10500.00  (4300-43200) | 11650.00  (3500-43200) | 10800.00  (3500-45400) | 7950.00  (2800-39400) | 8000.00  (3700-39400) | 0.076 |
|  | F | 12000.00  (3000-33600) | 12250.00  (3300-50600) | 11150.00  (3300-59400) | 11350.00  (2700-49700) | 10700.00  (2500-26900) | **b-e 0.001** |
| Platelets  ( x10^3^/mm^3^) | M | 203000  (124000-257000) | 209000  (152000-269000) | 198500  (124000-306000) | 198000  (106000-338000) | 197000  (101000-280000) | 0.473 |
|  | F | 215000  (162000-277000) | 202000  (160000-309000) | 205000  (178000-287000) | 202000  (154000-320000) | 189000  (125000-321000) | 0.998 |
| Hematocrite (%) | M | 43.00  (38.50-49.80) | 44.40  (36.70-49.80) | 44.15  (37.30-48.40) | 44.25  (38.00-49.50) | 42.80  (36.00-49.20) | 0.090 |
|  | F | 41.90  (38.90-44.90) | 41.20  (36.10-46.00) | 42.50  (36.40-45.70) | 41.15  (35.80-46.80) | 41.30  (36.00-47.10) | 0.190 |
| Hemoglobin (g/dL) | M | 14.80  (12.90-16.30) | 14.65  (12.20-16.30) | 14.90  (12.40-15.80) | 14.65  (12.50-16.00) | 14.10  (12.00-16.50) | 0.179 |
|  | F | 14.00  (12.80-15.70) | 13.70  (12.10-15.10) | 14.15  (12.00-15.60) | 13.95  (11.50-15.70) | 13.80  (11.70-16.10) | 0.110 |
| Monocytes  (x103/mm3) | M | 0.20  (0.20-1.00) | 0.55  (0.10-0.90) | 0.35  (0.10-1.30) | 0.35  (0.20-1.20) | 0.40  (0.10-1.10) | 0.350 |
|  | F | 0.35  (0.10-0.90) | 0.50  (0.10-2.00) | 0.55  (0.10-2.10) | 0.35  (0.20-1.60) | 0.30  (0.10-1.20) | 0.536 |
| Neutrophils  (x103/mm3) | M | 3.40  (1.70-7.50) | 5.03  (2.20-8.90) | 4.35  (1.70-6.60) | 3.60  (2.00-6.92) | 3.70  (1.60-5.90) | **b-e* 0.037** |
|  | F | 4.70  (2.50-5.80) | 4.95  (2.60-8.30) | 4.20  (2.20-8.00) | 4.25  (1.80-5.70) | 4.05  (2.10-6.80) | 0.095 |
| Eosinophils  (x103/mm3) | M | 0.21  (0.05-0.61) | 0.21  (0.06-0.48) | 0.24  (0.06-0.54) | 0.17  (0.06-0.43) | 0.17  (0.07-0.60) | 0.937 |
|  | F | 0.18  (0.09-0.42) | 0.21  (0.09-0.64) | 0.14  (0.09-0.42) | 0.18  (0.10-0.44) | 0.25  (0.09-0.57) | 0.218 |
| Basophils  (x103/mm3) | M | 0.04  (0.00-0.21) | 0.10  (0.00-0.21) | 0.05  (0.01-0.25) | 0.03  (0.01-0.10) | 0.02  (0.01-0.12) | 0.484 |
|  | F | 0.03  (0.01-0.36) | 0.07  (0.01-1.30) | 0.04  (0.01-0.14) | 0.04  (0.02-0.23) | 0.03  (0.00-0.10) | 0.082 |

* Friedman test indicates that there are significant differences at all time points of the intervention. However, Dunn-Bonferroni post hoc tests indicate that there are no significant pairwise differences.

**Table S2**. Differences in biochemical markers of patients with CLL (N=22) before and during the DI2 with High OC/OL**-**EVOO (M: male, F: female)

| **Biochemical Marker** | **Sex** | **6 months before**  **a**  **Median**  **(min-max)** | **3 months before**  **b**  **Median**  **(min-max)** | **Baseline**  **c**  **Median**  **(min-max)** | **3 months after**  **d**  **Median**  **(min-max)** | **6 months after**  **e**  **Median**  **(min-max)** | **p-value (adj)** |
| --- | --- | --- | --- | --- | --- | --- | --- |
| Glucose  (mg/dL) | M=12 | 103.00  (86.00-123.00) | 101.50  (86.00-138.00) | 105.50  (62.00-132.00) | 99.50  (58.00-125.00) | 98.00  (74.00-114.00) | 0.023***** |
|  | F=10 | 104.00  (93.00-123.00) | 104.00  (90.00-133.00) | 104.00  (88.00-128.00) | 100.00  (91.00-114.00) | 101.00  (77.00-120.00) | 0.053* |
| Urea  (mg/dL) | M | 44.00  (34.00-68.00) | 49.00  (31.00-83.00) | 49.00  (32.00-58.00) | 41.00  (34.00-64.00) | 44.00  (31.00-58.00) | 0.615 |
|  | F | 40.00  (25.00-55.00) | 39.50  (30.00-63.00) | 41.50  (30.00-85.00) | 39.00  (32.00-76.00) | 36.50  (30.00-60.00) | 0.053 |
| Uric Acid  (mg/dL) | M | 5.80  (3.00-8.30) | 5.90  (2.80-7.10) | 6.40  (3.20-53.00) | 6.25  (3.10-8.60) | 6.00  (2.90-8.50) | 0.353 |
|  | F | 5.25  (3.30-5.50) | 4.85  (3.20-6.20) | 4.85  (3.20-8.00) | 4.90  (3.20-5.80) | 4.55  (2.80-6.70) | 0.209 |
| Creatinin  (mg/dL) | M | 1.10  (0.90-1.20) | 1.00  (0.90-1.30) | 1.00  (0.70-1.20) | 0.90  (0.70-1.40) | 0.90  (0.80-1.45) | 0.006* |
|  | F | 0.80  (0.70-1.10) | 0.75  (0.60-1.40) | 0.80  (0.60-1.40) | 0.80  (0.70-1.30) | 0.80  (0.60-1.30) | 0.889 |
| SGPT  (U/L) | M | 19.00  (11.00-66.00) | 21.50  (8.00-46.00) | 21.50  (8.00-34.00) | 24.00  (10.00-74.00) | 15.00  (8.00-28.00) | 0.058 |
|  | F | 18.50  (14.00-20.00) | 19.00  (13.00-37.00) | 17.50  (13.00-27.00) | 20.00  (11.00-29.00) | 16.50  (12.00-35.00) | 0.796 |
| SGOT  (U/L) | M | 25.00  (14.00-51.00) | 23.50  (14.00-37.00) | 27.00  (14.00-40.00) | 26.00  (17.00-47.00) | 24.00  (18.00-29.00) | 0.113 |
|  | F | 23.50  (17.00-42.00) | 27.50  (13.00-54.00) | 23.00  (13.00-40.00) | 21.00  (11.00-40.00) | 22.00  (12.00-38.00) | 0.522 |
| LDH  (IU/L) | M | 213.00  (104.00-616.00) | 223.50  (104.00-576.00) | 205.00  (128.00-596.00) | 241.00  (164.00-445.00) | 180.00  (155.00-308.00) | 0.288 |
|  | F | 200.50  (169.00-308.00) | 208.00  (170.00-288.00) | 224.50  (173.00-313.00) | 208.50  (165.00-280.00) | 233.00  (150.00-641.00) | 0.622 |
| γGT  (U/L) | M | 19.00  (12.00-38.00) | 17.00  (11.00-45.00) | 16.00  (11.00-39.00) | 18.50  (13.00-37.00) | 15.00  (11.00-39.00) | 0.114 |
|  | F | 13.50  (10.00-21.00) | 14.00  (9.00-19.00) | 14.50  (9.00-24.00) | 14.00  (10.00-20.00) | 14.50  (7.00-30.00) | 0.537 |
| ALP  (U/L) | M | 73.00  (49.00-88.00) | 71.00  (54.00-240.00) | 70.50  (51.00-220.00) | 81.00  (52.00-198.00) | 77.00  (48.00-198.00) | 0.365 |
|  | F | 71.00  (47.00-90.00) | 67.50  (38.00-89.00) | 61.50  (46.00-89.00) | 78.50  (42.00-92.00) | 72.50  (34.00-84.00) | 0.060 |
| ***Lipidemic Profile*** |  |  |  |  |  |  |  |
| Total cholesterol (mg/dL) | M | 180.00  (137.00-208.00) | 178.00  (137.00-208.00) | 174.50  (141.00-195.00) | 184.00  (125.00-215.00) | 181.00  (125.00-203.00) | 0.918 |
|  | F | 220.00  (190.00-277.00) | 215.50  (162.00-255.00) | 220.00  (140.00-305.00) | 233.00  (162.00-300.00) | 197.50  (159.00-286.00) | 0.49 |
| Triglycerides  (mg/dL) | M | 106.00  (46.00-221.00) | 123.50  (49.00-221.00) | 126.00  (63.00-217.00) | 113.00  (45.00-191.00) | 90.00  (69.00-194.00) | 0.400 |
|  | F | 117.00  (84.00-250.00) | 97.50  (65.00-256.00) | 106.00  (65.00-299.00) | 109.00  (61.00-298.00) | 103.50  (67.00-280.00) | 0.838 |
| HDL cholesterol (mg/dL) | M | 57.00  (43.00-78.00) | 51.00  (37.00-70.00) | 54.50  (44.00-63.00) | 51.50  (32.00-62.00) | 50.00  (35.00-63.00) | 0.886 |
|  | F | 79.00  (34.00-92.00) | 60.00  (36.00-108.00) | 62.00  (33.00-99.00) | 59.00  (16.00-98.00) | 58.50  (35.00-82.00) | 0.084 |
| LDL cholesterol (mg/dL) | M | 102.00  (72.00-148.00) | 104.00  (70.00-148.00) | 93.00  (70.00-116.00) | 102.50  (73.00-139.00) | 105.00  (73.00-114.00) | 0.496 |
|  | F | 117.00  (100.00-185.00) | 122.50  (75.00-182.00) | 112.00  (68.00-212.00) | 137.50  (75.00-190.00) | 114.50  (68.00-200.00) | 0.139 |

***** Friedman test indicates that there are significant differences at all time points of the intervention. However, Dunn-Bonferroni post hoc tests indicate that there are no significant pairwise differences.

**Table S3.** Differences in apoptosis markers of patients with CLL (N=22) during the DI2 with High OC/OL**-**EVOO (M: male, F: female)

| **Apoptotic Markers** | **Sex** | **Baseline**  **a**  **Median**  **(min-max)** | **3 months**  **b**  **Median**  **(min-max)** | **6 months**  **c**  **Median**  **(min-max)** | **p-value**  **(adj)** |
| --- | --- | --- | --- | --- | --- |
| ccK18  (U/L) | M | 123.06  (85.31-400.45) | 142.61  (68.12-585.58) | 239.29  (147.00-335.00) | 0.078 |
|  | F | 131.26  (40.39-218.47) | 126.70  (46.94-385.24) | 184.44  (76.50-385.50) | 0.150 |
| Apo1-Fas  (U/L) | M | 83.57  (53.33-202.31) | 89.68  (66.01-230.18) | 140.87  (70.00-295.11) | **a-c 0.004** |
|  | F | 95.69  (66.99-141.00) | 95.36  (74.03-611.81) | 139.10  (73.00-855.41) | **a-c 0.042** |
| Survivin/API4 (pg/mL) | M | 187.34  (62.65-300.71) | 100.69  (62.13-181.89) | 75.00  (35.00-110.20) | **a-c 0.000**  **b-c 0.017** |
|  | F | 117.57  (81.00-429.50) | 109.00  (102.63-256.58) | 88.69  (55.00-125.00) | 0.071 |
